# Plate osteosynthesis versus hemiarthroplasty in proximal humerus fractures – Does routine screening of systemic inflammatory biomarkers makes sense?

**DOI:** 10.1186/s40001-014-0079-z

**Published:** 2015-01-14

**Authors:** Klemens Horst, Frank Hildebrand, Roman Pfeifer, Karin Köppen, Philipp Lichte, Hans-Christoph Pape, Thomas Dienstknecht

**Affiliations:** Department of Orthopaedics and Trauma Surgery, Aachen University Medical Centre, Pauwelsstrasse 30, 52074 Aachen, Germany

**Keywords:** CRP, Infection, Perioperative monitoring, Plate fixation, Proximal humerus fracture, Shoulder arthroplasty, WBC

## Abstract

**Background:**

Increases in C-reactive protein (CRP) and white blood cell (WBC) counts after orthopedic surgical procedures can give evidence of postoperative infection. However, there is a lack of knowledge about the kinetics of these biomarkers in cases with an uneventful clinical course after osteosynthesis of upper limb fractures. This study investigated CRP and WBC serum levels after osteosynthesis or hemiarthroplasty of humeral head fractures.

**Methods:**

A retrospective study on patients with humeral head fractures who had open reduction and internal fixation via plate osteosynthesis (PO) (*n* = 64) or hemiarthroplasty (HA) (*n* = 28) without any complications in the postoperative clinical course. C-reactive protein serum levels (mg/l) and leukocyte counts (g/l) were assayed at several time points. Multiple regression analysis was performed to evaluate the influence of several confounding variables (the surgical procedure, duration of surgery, patient’s health status, and comorbidities) on the kinetics of CRP and WBC.

**Results:**

Our data showed that CRP levels were statistically significantly higher in the HA cohort when compared to the PO cohort (*p* = 0.003). Moreover, daily measurement of CRP levels during the postoperative course showed that CRP peaked on the 2nd and 3rd days postoperatively in both cohorts and started to decrease afterward, reaching normal values on day 8 to 10. However, WBCs did not show any significant differences between the HA and PO cohorts. Finally, the choice of surgical procedure and the patient’s health status were associated with higher peak levels of CRP.

**Conclusions:**

After osteosynthesis or hemiarthroplasty of humeral head fractures, CRP is a responsive serum parameter in the postoperative course of an uneventful inflammatory response. Abnormalities from these values should be interpreted carefully as they may give a hint as to postoperative complications such as infection.

## Background

The incidence of humeral head fractures is increasing rapidly [1,2], especially in the elderly female population [2]. Several factors associated with humerus fractures have been identified: fragile bones and a patient at specific risk of falls. Operative treatment is frequently necessary but complication rates are high and increase with the degree of fracture severity [3-6]. Typical complications vary from malreduction to loss of anatomic fracture fixation, screw perforation, rotator cuff failure, infections, and delayed healing [7,8]. After surgical treatment of humeral head fractures, several complications have been identified either from the surgical technique (malreduction, perforating screws) or during the clinical course, especially postoperative infections [9,10].

C-reactive protein (CRP), an acute-phase protein, is known as a useful biomarker in detecting infections postoperatively [11-13]. Furthermore, the kinetics of systemic CRP levels have been associated with the course of infectious complications [14-17]. In addition, uneventful postoperative courses show a temporary increase in CRP levels [18-21]. For these reasons, the postoperative kinetics of systemic CRP concentrations need further investigation to differentiate between elevation related to a surgical procedure and elevation associated with infection in the postoperative course. Previous studies have investigated changes in CRP levels after diverse orthopedic procedures [22-24] as well as the leukocyte kinetics [25,26]. However, the kinetics of systemic CRP and WBC depend on both the severity and type of surgical procedure (e.g., tissue damage) as well as patient-related circumstances (e.g., health status, comorbidities) [22-24]. Therefore, investigating these effects in relation to specific anatomical regions and different operative procedures is important.

In the current study, we aimed to assess the kinetics of routine laboratory markers (CRP, WBC) after different surgical treatments of humeral head fractures. Additionally, we assessed the degree to which postoperative CRP/WBC kinetics are influenced by the patient’s comorbidities and perioperative status.

## Methods

### Patient enrolment

This retrospective study ran from 1 January 2010 to 31 December 2012, during which time 125 patients needed surgical treatment for proximal humerus fractures at Aachen University Medical Centre. Patients eligible for enrollment in the study presented with isolated humeral head fracture and were treated operatively using either plate osteosynthesis (PO) or hemiarthroplasty (HA). As no evidence-based recommendations on the treatment of proximal humerus fracture can be derived from the currently available data, the decision for either PO or HA was made in regard to the patients individual characteristics (biological age and bone quality, accompanying illnesses, compliance) and needs as recommended by Burkhart et al. and others [27-29]. Reasons for ineligibility were staged procedures or a history of autoimmune or inflammatory disorders, liver disease (including hepatitis), cancer, infectious complications after surgery, or postoperative antibiotic use.

### Clinical data

Laboratory results and other demographic data: comorbidities (diabetes mellitus, nicotine use, and alcohol misuse), ASA score, duration of the surgical procedure (from skin incision to closure), use of perioperative antibiotics, and the total length of stay in the hospital were collected from each patient’s chart.

### Analysis of inflammation biomarkers

Plasma levels of CRP and WBC were documented before surgery (at the time of admission) and on days 2 to 3, 4 to 5, 6 to 7, and 8 to 10 after the surgical procedure. Serum CRP was quantified by the Cobas 8000 modular analyzer series (Roche), while WBC was obtained using TS-2000 (Sysmex). CRP was reported in mg/l and WBC in g/l.

### Surgery

All surgical procedures were performed under general anesthesia. Perioperative antibiotic medication was cefuroxim 1.5 g (single shot) or clindamycin 1.5 g in cases of known allergic reaction to cefuroxim. A deltopectoral approach was used. Sutures were set into the insertions of the subscapularis, supraspinatus, and infraspinatus tendon. The humeral head was exposed to optimize visualization during the reduction procedure. Reduction was performed using digital pressure and retractors. Reduction of the tuberositas was performed. The result was held by temporary k-wires. The plate was attached to the humeral shaft with a bicortical screw inserted through the elongated hole. The plate position was checked by fluoroscopy and optimized before the rest of the screws had been inserted. Angular stable as well as normal plates were used. Finally, the rotator cuff tendons were secured via the sutures that were brought through the small holes in the plate.

Performing hemiarthroplasty, tendon sutures were set as described above. The humeral head was exposed by dividing the soft tissues over the fracture. The remaining medial capsular attachment to the head was released, and all fragments were removed. The humeral head was kept for later size measurements. The glenoid fossa was inspected, and the tuberositas were prepared. The medullary canal was opened and enlarged with rasps of increasing sizes. Humeral retroversion was measured and humeral head size determined. In the following step, the prosthesis was inserted (EPOCA, Synthes), the joint was reduced, and the tuberositas were fixated. Wound closure and final radiographic visualization as well as range of motion were verified at the end of the procedures.

### Statistical analysis

The statistical analysis was performed using the Statistical Package for Social Sciences (SPSS) (version 21.0.0.0). Variables such as age, body weight, BMI, duration of surgery, hospital length of stay, and peak values of CRP and WBC are reported as the mean and standard deviation (SD). The Kolmogorov-Smirnov test was performed to test for normal distribution. Student’s *t*-test and paired *t*-tests were used when applicable. The Mann–Whitney *U*-test was used if values were not normally distributed. Categorical variables are presented as frequencies (relative), and heterogeneities between the groups were assessed by chi-square tests. To assess the effect of the treatment modality on the peak values of the inflammatory parameters with adjustment for potential confounding factors, we used multifactorial linear regression models. *P* < 0.05 was considered statistically significant for all analyses.

## Results

### Demographics and clinical outcomes of cohorts

The overall study cohort consisted of 125 patients who were admitted to our hospital for humeral fracture fixation. Of these patients, 92 met our selection criteria (see Methods). Sixty-four patients had plate osteosynthesis (PO), and 28 had hemiarthroplasty (HA). Interestingly, our data showed that the HA cohort had a statistically significantly higher BMI (*P* = 0.013), longer operation time (*P* = 0.001), and longer hospital length of stay (*P* = 0.001) compared to the PO cohort. There was no statistical difference in age between the groups, but a trend toward an older population in the HA cohort (*P* = 0.051) (Table 1).

**Table 1:** General patient characteristics, comorbidities, and ASA-classification, * = p <0.05 (ASA-classification: American Society of Anaesthesiologists classification)

	***Plate osteosynthesis***	***Hemiarthroplasty***	***p-value***
	***(n = 64)***	***(n = 28)***	
***General characteristics***			
Age	64 ± 17	71 ± 12	0.051
Gender (female)	46 (73%)	18 (64%)	0.459
Body weight (kg)	73 ± 16	82 ± 21	0.026*
BMI	26 ± 5	29 ± 7	0.013*
BMI >25	30 (47%)	17 (61%)	0.208
Duration of Surgery (min)	111 ± 50	150 ± 57	0.001*
Duration of Surgery >2 h (n)	24 (37.5%)	20 (71%)	0.003*
Hospital length of stay (days)	7 ± 4	11 ± 6	0.001*
***Comorbidities***			
Nicotine	6 (9%)	1 (4%)	0.337
Alcohol	4 (6%)	0 (0%)	0.179
Diabetes mellitus	11 (17%)	5 (18%)	0.938
***ASA***			
I	3 (5%)	1 (4%)	0.810
II	42 (66%)	14 (50%)	0.160
III	20 (31%)	12 (43%)	0.285
IV	0 (0%)	1 (4%)	0.131
V	0 (0%)	0 (0%)	N/A

### Kinetics of CRP

**Figure 1:**
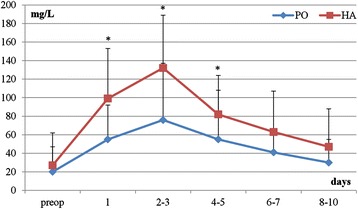
Kinetics of postoperative CRP levels after plate osteosynthesis (PO) vs. hemiarthroplasty (HA), *p < 0.05 on days 1, 2–3 and 4–5.

**Figure 2:**
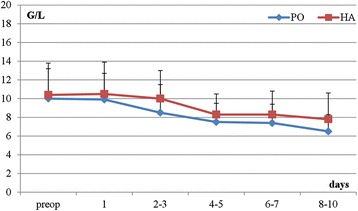
**Kinetics of postoperative WBC levels: plate osteosynthesis (PO) vs. hemiarthroplasty (HA).**

We measured CRP in a continuous fashion, preoperatively (time of admission), and then from day 1 up to 10 days postoperatively. Our data showed that preoperative values of CRP were increased in both groups (Table 2). Interestingly, the HA and PO groups had significant increases in CRP during the postoperative phase when compared to baseline (HA, *p* = 0.001; PO, *p* = 0.001) (Figures 1 and 2). Moreover, the highest CRP levels were observed between the 2nd and 3rd postoperative days in both groups. Indeed, there was a statistically significant difference in the peak values between the HA and PO groups (*p* = 0.010), where HA had higher levels of CRP. Finally, our analysis showed that there was a continuous decrease in CRP values over time till the baseline levels were reached between the 8th and 10th postoperative days (Table 2, Figure 1).

**Table 2:** Mean profiles of CRP and WBC values pre- and postoperatively, * = p <0.05

	***Plate osteosynthesis***	***Hemiarthroplasty***	***p-value***
	***(n = 64)***	***(n = 28)***	
***CRP (mg/L)***			
Preoperatively	20 ± 27	27 ± 35	0.387
Day 1	55 ± 37	99 ± 54	0.003*
Day 2-3	76 ± 61	132 ± 57	0.003*
Day 4-5	55 ± 53	82 ± 42	0.013*
Day 6-7	41 ± 23	63 ± 44	0.119
Day 8-10	30 ± 25	47 ± 41	0.513
***WBC (G/L)***			
Preoperatively	10.0 ± 3.4	10.4 ± 3.2	0.248
Day 1	9.9 ± 3.4	10.5 ± 2.8	0.319
Day 2-3	8.5 ± 3.0	10.0 ± 3.0	0.137
Day 4-5	7.5 ± 2.2	8.3 ± 2.0	0.113
Day 6-7	7.4 ± 2.5	8.3 ± 2.0	0.423
Day 8-10	6.5 ± 2.8	7.8 ± 1.8	0.438

### Kinetics of WBC

We measured WBC counts in the same fashion as CRP. Our analysis showed no statistical difference in WBC counts in the preoperative phase when compared to the baseline. In addition, there was no statistical difference in WBC counts between the HA and PO groups over time in the postoperative course. Finally, we observed a continuous decrease in WBC counts after the surgical intervention, with the lowest counts at the end of the observation time (*p* = 0.051) (Table 2, Figure 2).

### Influence of the surgical procedure and patient’s health status

Multiple regression analysis was carried out to assess correlations between CRP and WBC peak values and the characteristics of both surgical procedures [surgical approach used and duration of surgery) as well as the general health status (American Society of Anesthesiologists risk classification (ASA) and body mass index (BMI)] of the patients. In this context, the ASA classification and surgical approach were significantly correlated with peak CRP levels (Table 3). However, there was no significant correlation with the duration of the surgical intervention and BMI (Table 3). On the other hand, postoperative WBC peaks did not show any correlation with the parameters we assessed (Table 3). Furthermore, we tested whether preoperative parameters such as age and ASA might predict the choice of surgical approach but did not find a significant correlation.

**Table 3:** Multiple regression analysis of peak CRP and WBC values, * = p <0.05

	***Peak CRP***	***Peak WBC***
	***PO vs. HA***	***PO vs. HA***
**Model Summary**		
Adjusted R^2^	0.22	0.07
**ANOVA**		
F-value	7.2	2.6
*p*-value	<0.001*	0.041*
**Coefficients**		
Constant (SE)	−75.35 (35.11)	8.43 (2.11)
*p*-value	0.035*	<0.001*
ASA	29.15 (10.36)	−0.85 (0.62)
*p*-value	0.006*	>0.05
Surgical procedure	40.15 (14.32)	0.97 (0.86)
*p*-value	0.006*	>0.05
Surgical duration	0.072 (0.12)	0.01 (0.01)
*p*-value	>0.05	>0.05
BMI	1.36 (1.04)	0.07 (0.06)
*p*-value	>0.05	>0.05

### Comorbidities

The prevalences of comorbidities between the HA and PO cohorts were comparable (Table 1). Multiple regression analysis showed that CRP and WBC peaks were not correlated with any comorbidities we observed in our patients.

## Discussion

Humeral head fracture is common in all age groups [1,30,31], particularly in aged patients. In addition, being female increases the odds [1,2,30]. Falls and motor vehicle accidents (MVA) can cause humeral fractures [31]. However, while different surgical approaches can be used in treating such fractures, controversy still remains over which should be chosen: plate osteosynthesis (PO), hemiarthroplasty (HA), or total arthroplasty (TSA). [29]. Outcomes of treating humeral fractures depend on both the patient’s health status and the severity of the fracture, along with the surgeon’s experience [29]. Moreover, technical problems (screw perforation and malreduction) and postoperative complications (infection) in the clinical course can affect the outcome. Surgical intervention affects the inflammatory response, especially in the early phase [32]. Certain complications may occur during this critical phase, of which wound infections are of high importance. However, lack of knowledge about the proper postoperative inflammatory response after surgical intervention for humeral head fractures can lead to overlooked complications or misdiagnosis [7,10]. In this context, CRP is an acute phase protein that can be used as a marker for changes in the orthopedic postoperative inflammatory response. Yet, it is a necessary prerequisite to know the kinetics of CRP in the case of an uneventful postoperative course [32]. Our analysis showed that CRP peaks on the 2nd to 3rd day after the PO or HA procedure. Moreover, both surgical approaches affect CRP values regardless of the duration of the surgery as well as the patient’s health status. However, our analysis did not show any comorbidities in our patients that affected CRP values.

### CRP kinetics in orthopedic trauma

The increase in preoperative CRP values in our data can be explained by the well-known early elevation of systemic CRP levels after tissue damage [33,34]. The postoperative course is affected by age, the type of surgical approach, and operated body region [22-24,32,35-37]. In the field of orthopedic trauma, several studies have examined the effect of different surgical approaches to hip fracture [23,38,39]. Neumaier et al. studied the CRP kinetics after surgery in different body regions. However, regarding the postoperative CRP kinetics of the humerus, the author did not distinguish between surgery of the proximal humerus, the shaft or the distal part of the humerus. Also no difference was reported in diverse surgical procedures regarding humerus fractures [32]. Moreover, they did not find an effect of the particular surgical approach on peak CRP levels. In this context, our analysis showed that CRP levels rise to their maximum amplitude on the 2nd to 3rd postoperative day with both the HA and PO approaches. Furthermore, peak levels in HA patients were significantly higher than those in patients with the PO approach, but showed an equivalent decrease after reaching their peak. These patterns were comparable to previously published reports comparing osteosynthesis versus arthroplasty and reflect the patient’s recovery [22,23]. In contrast to this course of CRP after uneventful orthopedic surgery, a secondary increase or a persistent elevation in cases where infectious complications develop has been described [22].

### Influence of surgical approach on CRP kinetics

The severity of the tissue damage, type of tissue (fat, muscle, or bone), and traumatized body region have all been shown to influence postoperative CRP kinetics [22,23,40,41]. Most previous studies have focused on CRP kinetics after operative treatment of lower limbs (femur and knee) or the vertebral column after trauma [22,24,41]. However, our study revealed CRP kinetics after uneventful operative treatment of humeral head fractures by comparing two different approaches to surgical treatment. Our results suggest that the HA approach is associated with higher CRP peaks when compared to the PO approach.

In conclusion, our findings support the proposition that high CRP concentrations correlate positively with increased damage to muscles and bone caused by removing the humeral head, using a reamer to prepare the intramedullary canal and cementing the prosthesis [41,42].

### Comorbidities and CRP kinetics

The literature regarding several affecting factors (type of operation, patient health status, and demographic-related factors) is contradictory [43]. Larsson et al. reported that the type of anesthesia, amount of bleeding, transfusion, operation time, administered drugs (antibiotics), age, and gender did not have any influence on peak CRP levels. On the other hand, other studies showed that the duration of surgery, obesity, and the use of anesthetics influence postoperative immunologic reactions [24,38,44]. In contrast to Kraft et al., our data show that a patient’s health status as measured by the ASA classification is positively correlated with CRP peaks [24], because the population investigated by Kraft et al. experienced different surgical interventions and was younger than ours. In this context, as age is known to be associated with a significant reduction in a patient’s overall health status, we conclude that a reduced ASA classification may contribute to higher CRP peaks postoperatively. This observation could not be proven for single comorbidities such as obesity, alcohol or nicotine abuse, and diabetes mellitus, strengthening the proposition to consider the patient’s overall health status rather than single comorbidities when evaluating postoperative CRP.

### Leukocyte kinetics

In accordance with previously published studies, we observed a postoperative decrease in WBC counts [24,45]. Moreover, there was no significant difference in the WBC counts or in their peak levels between the HA and PO cohorts. This difference in the kinetics of WBC and CRP is explainable, as CRP shows an individual stability and narrow normal range, is barely influenced by common comorbidities (except liver diseases), and shows distinctive patterns with different surgical approaches. In conclusion, measuring CRP is more informative than WBC in detecting any unusual changes in the early postoperative phase [24,46].

### Limitations

We recognize that there are several limitations in our study. First, this study was a retrospective study performed at a single trauma center, and thus it may not be generalizable or pertinent to other centers with differing admission demographics, injury characteristics, or management practices. Also, it would have been interesting to gain information about the kinetics of advanced inflammatory parameters (e.g., IL-6). Moreover, we had limited patient numbers. In general, younger patients and non-displaced or mildly displaced fractures are treated conservatively, while the treatment of choice for displaced proximal humerus fractures is anatomical reconstruction and osteosynthesis. In the elderly, the implantation of a prosthesis may need to be considered in order to restore painless, robust function of the humerus, and thus personal independence, as rapidly as possible. Information about individual aspects that led to the decision to employ HA or PO was lacking. However, studies regarding threshed values for either one surgical technique are sparse and should gain more attention as guidelines are necessary. Finally, our study was restricted to a specific injury and operative approaches, and detailed information about wound length as another indicator for surgical trauma was not documented (HA vs. PO).

## Conclusions

In conclusion, the current study demonstrates unique inflammatory biomarker patterns, particularly in the early events post-injury, which emerge in humeral head fracture patients, suggesting that CRP can potentially predict or help in diagnosing postoperative complications. In the context of the presented data and literature we suggest routine measurement of serum CRP levels in a continuous fashion from the day of admission, 2nd to 3rd, and 4th to 5th day postoperatively, as secondary relapse may help in detecting postoperative infection [22,32]. If so, screening for the source of infection (e.g., wound infection, pneumonia, urinary tract infection) should start immediately. Finally, further studies are needed to validate the sufficiency of the time intervals we suggest for early detection of infectious complications after proximal humerus fracture fixation.

## Abbreviations

HA: Hemiarthroplasty
PO: Plate osteosynthesis
CRP: C-reactive protein
WBC: White blood cells
BMI: Body mass index
ASA: American Society of Anesthesiologists
SD: Standard deviation

## References

[1] Palvanen M, Kannus P, Niemi S, Parkkari J (2006). Update in the epidemiology of proximal humeral fractures. Clin Orthop Relat Res.

[2] Kim SH, Szabo RM, Marder RA (2012). Epidemiology of humerus fractures in the United States: nationwide emergency department sample, 2008. Arthritis Care Res (Hoboken).

[3] Tepass A, Rolauffs B, Weise K, Bahrs SD, Dietz K, Bahrs C (2013). Complication rates and outcomes stratified by treatment modalities in proximal humeral fractures: a systematic literature review from 1970–2009. Patient Saf Surg.

[4] Roux A, Decroocq L, El Batti S, Bonnevialle N, Moineau G, Trojani C (2012). Epidemiology of proximal humerus fractures managed in a trauma center. Orthop Traumatol Surg Res.

[5] Jaeger M, Izadpanah K, Maier D, Reising K, Strohm PC, Sudkamp NP (2012). Fractures of the humerus head. Chirurg.

[6] Okike K, Lee OC, Makanji H, Harris MB, Vrahas MS (2013). Factors associated with the decision for operative versus non-operative treatment of displaced proximal humerus fractures in the elderly. Injury.

[7] Smith AM, Mardones RM, Sperling JW, Cofield RH (2007). Early complications of operatively treated proximal humeral fractures. J Shoulder Elbow Surg.

[8] Braunstein V (2013). Proximal humerus fractures. Decisive factors for therapy choice, treatment and complications. Unfallchirurg.

[9] Athwal GS, Sperling JW, Rispoli DM, Cofield RH (2007). Acute deep infection after surgical fixation of proximal humeral fractures. J Shoulder Elbow Surg.

[10] Blonna D, Barbasetti N, Banche G, Cuffini AM, Bellato E, Masse A (2014). Incidence and risk factors for acute infection after proximal humeral fractures: a multicenter study. J Shoulder Elbow Surg.

[11] Tillett WS, Francis T (1930). Serological reactions in pneumonia with a non-protein somatic fraction of pneumococcus. J Exp Med.

[12] Carr WP (1983). The role of the laboratory in rheumatology. Acute-phase proteins. Clin Rheum Dis.

[13] Pepys MB (1981). C-reactive protein fifty years on. Lancet.

[14] Peltola H, Vahvanen V, Aalto K (1984). Fever, C-reactive protein, and erythrocyte sedimentation rate in monitoring recovery from septic arthritis: a preliminary study. J Pediatr Orthop.

[15] Sabel KG, Hanson LA (1974). The clinical usefulness of C-reactive protein (CRP) determinations in bacterial meningitis and septicemia in infancy. Acta Paediatr Scand.

[16] Mustard RA, Bohnen JM, Haseeb S, Kasina R (1987). C-reactive protein levels predict postoperative septic complications. Arch Surg.

[17] Verkkala K, Valtonen V, Jarvinen A, Tolppanen EM (1987). Fever, leucocytosis and C-reactive protein after open-heart surgery and their value in the diagnosis of postoperative infections. Thorac Cardiovasc Surg.

[18] Boralessa H, de Beer FC, Manchie A, Whitwam JG, Pepys MB (1986). C-reactive protein in patients undergoing cardiac surgery. Anaesthesia.

[19] Fischer CL, Gill C, Forrester MG, Nakamura R (1976). Quantitation of “acute-phase proteins” postoperatively. Value in detection and monitoring of complications. Am J Clin Pathol.

[20] Aalto K, Osterman K, Peltola H, Rasanen J (1984). Changes in erythrocyte sedimentation rate and C-reactive protein after total hip arthroplasty. Clin Orthop Relat Res.

[21] Stahl WM (1987). Acute phase protein response to tissue injury. Crit Care Med.

[22] Larsson S, Thelander U, Friberg S (1992). C-reactive protein (CRP) levels after elective orthopedic surgery. Clin Orthop Relat Res.

[23] Neumaier M, Metak G, Scherer MA (2006). C-reactive protein as a parameter of surgical trauma: CRP response after different types of surgery in 349 hip fractures. Acta Orthop.

[24] Kraft CN, Kruger T, Westhoff J, Luring C, Weber O, Wirtz DC (2011). CRP and leukocyte-count after lumbar spine surgery: fusion vs. nucleotomy. Acta Orthop.

[25] Hughes SF, Hendricks BD, Edwards DR, Maclean KM, Bastawrous SS, Middleton JF (2010). Total hip and knee replacement surgery results in changes in leukocyte and endothelial markers. J Inflamm (Lond).

[26] Yasmin D, Bulut G, Yildiz M (2006). Can procalcitonin be used for the diagnosis and follow-up of postoperative complications after fracture surgery?. Acta Orthop Traumatol Turc.

[27] Handoll HH, Ollivere BJ, Rollins KE (2012). Interventions for treating proximal humeral fractures in adults. Cochrane Database Syst Rev.

[28] Burkhart KJ, Dietz SO, Bastian L, Thelen U, Hoffmann R, Muller LP (2013). The treatment of proximal humeral fracture in adults. Dtsch Arztebl Int.

[29] Maier D, Jaeger M, Izadpanah K, Strohm PC, Suedkamp NP (2014). Proximal humeral fracture treatment in adults. J Bone Joint Surg Am.

[30] Singer BR, McLauchlan GJ, Robinson CM, Christie J (1998). Epidemiology of fractures in 15,000 adults: the influence of age and gender. J Bone Joint Surg (Br).

[31] Bercik MJ, Tjoumakaris FP, Pepe M, Tucker B, Axelrad A, Ong A (2013). Humerus fractures at a regional trauma center: an epidemiologic study. Orthopedics.

[32] Neumaier M, Scherer MA (2008). C-reactive protein levels for early detection of postoperative infection after fracture surgery in 787 patients. Acta Orthop.

[33] Foglar C, Lindsey RW (1998). C-reactive protein in orthopedics. Orthopedics.

[34] Husain T, KIM DH (2002). C-reactive protein and erythrocyte sedimentation rate in orthopaedics. Univ Pa Orthop J.

[35] Pinato DJ, Bains J, Irkulla S, Pomroy J, Ujam B, Gaze D (2013). Advanced age influences the dynamic changes in circulating C-reactive protein following injury. J Clin Pathol.

[36] Brewster N, Guthrie C, McBirnie J (1994). CRP levels as a measure of surgical trauma: a comparison of different general surgical procedures. J R Coll Surg Edinb.

[37] Thelander U, Larsson S (1992). Quantitation of C-reactive protein levels and erythrocyte sedimentation rate after spinal surgery. Spine (Phila Pa 1976).

[38] Niskanen RO, Korkala O, Pammo H (1996). Serum C-reactive protein levels after total hip and knee arthroplasty. J Bone Joint Surg (Br).

[39] Okafor B, MacLellan G (1998). Postoperative changes of erythrocyte sedimentation rate, plasma viscosity and C-reactive protein levels after hip surgery. Acta Orthop Belg.

[40] Scherer MA, Neumaier M, von Gumppenberg S (2001). C-reactive protein in patients who had operative fracture treatment. Clin Orthop Relat Res.

[41] Shen H, Zhang N, Zhang X, Ji W (2009). C-reactive protein levels after 4 types of arthroplasty. Acta Orthop.

[42] White J, Kelly M, Dunsmuir R (1998). C-reactive protein level after total hip and total knee replacement. J Bone Joint Surg (Br).

[43] Schumann R (2012). Obesity, surgery, and perioperative inflammation research: where is it going?. Bariatric times.

[44] Mahmoud K, Ammar A (2011). Immunomodulatory effects of anesthetics during thoracic surgery. Anesthesiol Res Pract.

[45] Codine P, Barbotte E, Denis-Laroque F, Lansac H, Dupetit T, Pradies F (2005). C-reactive protein, leukocyte count and D-dimer monitoring after orthopedic surgery: early diagnosis of infectious or thromboembolic complications. Part one: C-reactive protein and leukocyte count as an aid in diagnosing postoperative infection. Ann Readapt Med Phys.

[46] Pepys MB, Hirschfield GM (2003). C-reactive protein: a critical update. J Clin Invest.

